# Age- and Diet-Dependent Changes in Hepatic Lipidomic Profiles of Phospholipids in Male Mice: Age Acceleration in Cyp2b-Null Mice

**DOI:** 10.1155/2022/7122738

**Published:** 2022-03-29

**Authors:** Melissa M. Heintz, Ramiya Kumar, Kristal M. Maner-Smith, Eric A. Ortlund, William S. Baldwin

**Affiliations:** ^1^Clemson University, Biological Sciences, Clemson, SC, USA; ^2^Emory University School of Medicine, Atlanta, GA, USA

## Abstract

Increases in traditional serum lipid profiles are associated with obesity, cancer, and cardiovascular disease. Recent lipidomic analysis has indicated changes in serum lipidome profiles, especially in regard to specific phosphatidylcholines, associated with obesity. However, little work has evaluated murine hepatic liver lipidomic profiles nor compared these profiles across age, high-fat diet, or specific genotypes, in this case the lack of hepatic Cyp2b enzymes. In this study, the effects of age (9 months old), high-fat diet (4.5 months old), and the loss of three primarily hepatic xeno- and endobiotic metabolizing cytochrome P450 (Cyp) enzymes, *Cyp2b9*, *Cyp2b10*, and *Cyp2b13* (Cyp2b-null mice), on the male murine hepatic lipidome were compared. Hierarchical clustering and principal component analysis show that age perturbs hepatic phospholipid profiles and serum lipid markers the most compared to young mice, followed by a high-fat diet and then loss of Cyp2b. Several lipid biomarkers such as PC/PE ratios, PE 38 : 6, and LPC concentrations indicate greater potential for NAFLD and hypertension with mixed effects in Cyp2b-null mice(less NAFLD and greater hypertension-associated markers). Lipid profiles from older mice contain greater total and n-6 fatty acids than normal diet (ND)-fed young mice; however, surprisingly, young Cyp2b-null mice contain high n-6 : n-3 ratios. Overall, the lack of Cyp2b typically enhanced adverse physiological parameters observed in the older (9 mo) mice with increased weight gain combined with a deteriorating cholesterol profile, but not necessarily all phospholipid profiles were adversely perturbed.

## 1. Introduction

Obesity is a major risk factor for metabolic disorders such as cardiovascular disease, diabetes, and fatty liver disease. Data from the most recent National Health and Nutrition Examination Survey in 2015-2016 shows that 39.8% of adults and 18.5% of youth in the United States are obese [1]. Disease susceptibility and overall health is greatly affected by changes to the lipidome [2, 3]. High-fat diets, such as the Western diet, cause obesity and drastically alter the hepatic lipidome [4], and perturbed lipid profiles are associated with specific liver diseases, such as nonalcoholic fatty liver disease (NAFLD) and nonalcoholic steatohepatitis (NASH) [5, 6]. Age also alters the phospholipid profile of mitochondria in the liver, brain, and skeletal tissue [7, 8]. Age transcended the effect of a high-fat diet on alterations to the blood lipidome in female mice (males were not investigated) [9]; however, little is known about changes that occur with age to the hepatic lipidome.

Lipids provide membrane structure and energy storage and act as signaling molecules that mediate lipid metabolism, inflammation, and progression of chronic diseases such as insulin resistance [10]. For example, the polyunsaturated fatty acid (PUFA), linoleic acid, is the endogenous ligand for hepatic nuclear factor 4*α* (HNF4*α*), a key regulator of multiple metabolic pathways [11]. Several fatty acids are peroxisome proliferator-activated receptor (PPAR) ligands [12] and fatty acids released by lipolysis during fasting trigger hepatic PPAR*α*-mediated *β*-oxidation while inhibiting lipogenesis through the liver X receptor (LXR) [13]. During inflammation, PUFAs found in hepatic membrane phospholipids are cleaved by phospholipase A2 [14]. These available PUFAs are then oxidized by cyclooxygenase, lipoxygenase, or cytochrome P450s (CYP) to form physiologically significant metabolites. The CYP pathways typically metabolize PUFAs to fatty acid epoxides, called oxylipins, that have bioactive effects [15]. CYPs including CYP1A, CYP1B, CYP2B, CYP2C, CYP2D, CYP2J, CYP3A, CYP4A, and CYP4F all metabolize PUFAs [15–17].

Cyp2b enzymes are key detoxification enzymes as are most of the CYPs found in families 1-3 [18]. Cyp2b enzymes are involved in the metabolism of numerous environmental, pharmaceutical, and endobiotic chemicals including organophosphate pesticides, several neuroactive drugs, fatty acids, and steroids [19–25]. Mice with repressed Cyp2b expression such as RNAi-mediated Cyp2b-knockdown (KD) and Cyp2b-null show greater toxicity to several chemicals, including parathion, zoxazolamine, and perfluorooctanesulfonate (PFOS) [26, 27].

Our lab previously produced a Cyp2b9/10/13-null (Cyp2b-null) mouse model, lacking the primary hepatic Cyp2b members; Cyp2b9, Cyp2b10, and Cyp2b13 on a C57Bl/6J (B6) background [28] and the Cyp2b-null males are diet-induced obese (DIO) with development of NAFLD [29]. Similarly, human CYP2B6 is the only human detoxification CYP associated with obesity; low liver CYP2B6 expression is associated with obesity [30]. In addition, the Cyp2b-null males develop nonalcoholic steatohepatitis (NASH) after treatment with a choline-deficient amino acid defined high-fat diet methionine-choline-deficient (CDAHFD) diet probably because of increased NAFLD compared to WT mice [31]. However, Cyp2b-null females are protected from CDAHFD-mediated NASH and NAFLD in comparison to WT mice in association with lower inflammatory and diabetic markers [32]. Increased hepatic lipid accumulation was also observed in male Cyp2b-KD mice on a FVB/NJ background as the mice aged [26]. However, the hepatic phospholipid profile has not been investigated in Cyp2b-null mice. Phospholipids are predominantly synthesized in the liver and responsive to dietary changes [33, 34]. Phospholipids are also key molecules in the structure of cells, development, signal transduction, immune and inflammatory responses, repair, and lipoprotein synthesis, and profile changes are associated with metabolic disease [4, 35, 36]. The previously referenced studies with Cyp2b-KD and Cyp2b-null mice showed few adverse differences between genotypes in female mice, but significant differences in obesity in male mice, most likely because several strains of mice are less susceptible to obesity in females [37, 38]. Therefore, in this study, we compared the hepatic lipidome of male Cyp2b-null and WT mice in healthy, diet-induced obese, and older mice.

## 2. Materials and Methods

### 2.1. Treatment of Experimental Groups

Animal care procedures were approved by Clemson University's Institutional Animal Care and Use committee. Cyp2b-null mice were developed using CRISPR/Cas9 as previously described [28], and wild-type (WT) B6 mice were purchased from The Jackson Laboratory (Bar Harbor, ME, USA) at 3 weeks of age and acclimated for 6 weeks prior to treatment. WT and Cyp2b-null male (9 weeks old) mice were divided into groups (*n* = 9) and fed either commercially available diets, either a normal chow diet (ND; 2018S-Envigo Teklad Diet, 3.1 Kcal/g: 18.6% protein, 6.2% fat, and 44.2% carbohydrates; Madison, WI USA) or a high-fat diet (HFD; Envigo TD.06414, 5.1 Kcal/g: 60.3% fat (37% saturated, 47% monounsaturated, and 16% polyunsaturated fat), 18.4% protein, and 21.3% carbohydrates; Madison, WI USA) for 10 weeks [29]. Mice were 4.5 months old at the end of the HFD study and referred to as ND-fed young or HFD-fed young WT or Cyp2b-null mice. An additional experimental group of WT (Jackson) and Cyp2b-null male mice (*n* = 5) were fed a ND until they reached 9 months (termed old WT and old Cyp2b-null mice). At the end of the studies, mice were fasted 4 hours (from 08 : 00–12 : 00) and then weighed, anesthetized, and blood collected by heart puncture prior to euthanasia and serum preparation. Serum biomarkers and liver triglycerides (TAG) were measured as described [29]. Liver and inguinal white adipose tissue (WAT) were excised, weighed, and divided by total body weight to determine the hepatosomatic index (HSI) and white adipose somatic index (WSI). The tissues were immediately snap frozen in liquid nitrogen and stored at -80°C.

### 2.2. Standards

Synthetic standards were obtained from Avanti Polar Lipids (Alabaster, AL, USA) for use internal standards. These include di-17 : 0 phosphatidylcholine (PC) (*x* : *y* where *x* indicates number of carbons and y indicates number of double bonds in fatty acid constituents), di-17 : 0 phosphatidylethanolamine (PE), 17 : 0 lysophosphatidylcholine (LPC), and 17 : 0 lysophosphatidylethanolamine (LPE). Working concentrations of spiked internal standards were 0.91 mg/ml, 0.36 mg/ml, 0.36 mg/ml, and 0.91 mg/ml, respectively.

### 2.3. Lipid Extraction

For targeted lipidomics assays, liver samples were extracted using a modified Bligh and Dyer lipid extraction protocol [39], whereby a ratio of chloroform and methanol was used to ensure robust extraction of all major lipid classes. Briefly, 100 mg liver was homogenized in 500 *μ*l phosphate-buffered saline (PBS, pH 7.4). To the homogenate, 2 ml of methanol/chloroform (2 : 1 *v*/*v* ratio) was added, and the samples were vortexed to ensure homogeneity of sample. To aid in the complete recovery of zwitterionic lipids, 100 *μ*l 0.1 mM sodium chloride was added. The organic phase was recovered and dried under nitrogen gas, and the lipid weight was recorded. Recovered lipids were then reconstituted in 1 ml of 1 : 1 *v*/*v* chloroform/methanol prior to analysis by LC/MS.

### 2.4. Mass Spectrometry

Targeted lipidomics experiments were conducted using Sciex AC LC system and Sciex QTrap5500 mass spectrometer (Framingham, MA, USA). Phospholipid species were identified and quantified from the livers of mice (*n* = 3 for young (4.5 mo) ND- and HFD-fed mice; *n* = 5 for old (9 mo.) mice) from each experimental group by LC-MS/MS at the Emory Integrated Metabolomics and Lipidomics Core (EIMLC). Ten microliters of resolvated lipids were deposited onto Thermo Scientific Accucore C18 column (4.6 × 100 mm, 2.6 *μ*m) with a column temperature of 40°C and mobile phases of (A) 40 : 60 water/acetonitrile and (B) 90 : 10 isopropanol/acetonitrile both with 0.1% formic acid and 1 mM ammonium formate at a flow rate of 0.5 ml/min. Lipids were resolved on an 18-minute linear gradient using these solvents and gradients that are recorded in Table 1. Instrumental parameters, such as electrospray voltage, declustering potential, and collision energies, were optimized using the internal standard and held constant during the course of the experiment. Subsequent to the optimization of instrumental parameters, the linear range of detection was determined using the same synthetic standards. A table of instrumental parameters is shown in Table 2.

Polyunsaturated fatty acids (PUFA) were selectively targeted in extracted liver samples by performing precursor ion scans in the negative ion mode. These include linoleic acid (LA; 18 : 2), *α*-linolenic acid (ALA; 18 : 3), arachidonic acid (ARA; 20 : 4), and docosahexaenoic acid (DHA; 22 : 6). The resulting precursor ion scans, corresponding to the molecular weights, are m/z 279, m/z 277, m/z 303, and m/z 327, respectively. All peaks above signal to noise ratio of 5 were fragmented for identification. The area under the curve for all precursors were used to calculate relative percentages and used to compare changes in lipid distribution between cohorts. For total quantification, the area under the curve is calibrated against the area of an internal standard of known concentration.

### 2.5. Lipid Quantification

Relative percentages of targeted lipids were quantified by first summing all lipids with the same precursor and then by dividing individual species by that sum and multiplying that digit by 100. This allows for comparison of select lipids between subjects in a cohort. For total quantification, the area under the curve is calibrated against the area of an internal standard of known concentration. For this, the area of the lipid in question is multiplied by the concentration of the spiked standard and then divided by the area of the spiked standard. This single point calibration is used to determine molar concentration as well as to adjust for matrix affects not seen in the external calibration curve used to determine limits of detection and quantification. For annotation of quantified lipids, standard lipidomics nomenclature was used, where acyl linkages are standard and ether linked lipids were denoted as p = plasmalogen subclass and e = alkyl ether subclass. Aliphatic groups in lipid classes were also denoted as *x* : *y*, where *x* is the number of carbons and *y* is the number of double bonds (e.g., 20 : 4 (arachidonic acid) has 20 carbons and 4 double bonds).

### 2.6. Lipid Annotation

Raw lipidomics data was analyzed using dedicated Sciex instrument software Analyst 1.5 and LipidView. Analyst was used to visually inspect peaks to ensure reproducible retention times and peak shape and also to manually extract ions for confirmation of putative lipid identifications. LipidView, a lipid database containing over 25,000 lipid species from more than 50 lipid classes, was used to for putative lipid assignments. Lipid profiles were then created in Excel using peak areas to visualize changes in the abundance of lipid species. Raw signal, observed in counts per second, that are five times the background noise threshold are considered quality data and used to create profiles.

### 2.7. Statistical Analysis

Data are presented as mean ± SEM. Statistical significance was determined (*p* value < 0.05) by unpaired Student's *t*-tests when comparing two groups, a one-way ANOVA followed by Fisher's LSD as the post hoc test when comparing more than two groups with GraphPad Prism 7.0 (GraphPad Software, San Diego, CA, USA). Hierarchical cluster analysis was performed on lipidomic data and visualized in heat maps with MetaboAnalyst 3.6 [40] to compare lipid species content across treatment groups. Random forest (http://www.r-project.org/) was used to rank phospholipid species as a prediction of the significance of an effect each lipid species has on differences between treatment groups [41]. The tuneRF() function was used to determine the best number of predictor (mtry) value to get the lowest out-of-bag (OOB) classification error as trees are added to the forest. The number of trees to be built (ntree) was set to 350 for all experimental groups to achieve the lowest OOB error. The larger the mean decreased accuracy (MDA) value, the more important the phospholipid species are for the accuracy of the association between variable and response. Lipid species with importance scores less than or equal to zero are likely to have no predictive ability. Principal component analysis (PCA) was performed and a biplot drawn using the ggbiplot package in R to compare the relationship between multiple variables and treatment groups. Variables included total body weight, WSI, serum lipids, and phospholipid species. The factoextra R package (https://cran.r-project.org) was used to obtain the percent contributions of each measured variable in principle components 1-3.

## 3. Results

### 3.1. Increase in Obesity due to a High-Fat Diet or Age Is Exacerbated in Cyp2b-Null Mice

**Figure 1:**
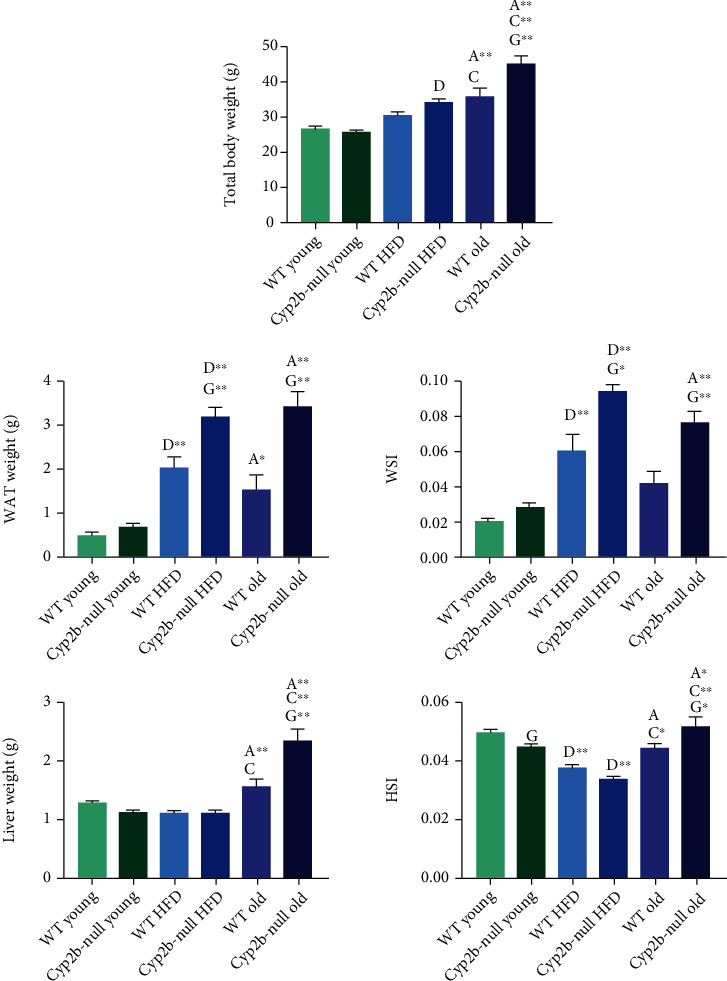
Comparison of total body, liver, and WAT weights between all treatment groups. Total body weight, liver weight, hepatic somatic index (HSI), WAT weight, and WAT somatic index (WSI) were measured for all treatment groups. Data are presented as mean (g) ± SEM. Statistical significance was determined by one-way ANOVA multiple comparison test with Tukey's multiple comparison test as the post hoc test (*n* = 5 − 9). “a” (age) indicates age difference between young (4.5 mo) and old (9 mo) mice within the same genotype and diet group, “c” (catch) indicates difference between HFD-fed young (4.5 mo) and ND-fed old (9 mo) mice within same genotype, “d” (diet) indicates diet difference between ND-fed and HFD-fed mice within in same genotype and age, and “g” (genotype) indicates genotype difference between WT and Cyp2b-null mice within same diet and age group. No asterisk indicates a *p* value < 0.05, ∗ indicates a *p* value < 0.01, and ∗∗ indicates a *p* values < 0.0001.

ND-fed old Cyp2b-null male mice weigh more than all other groups (Figure 1). Cyp2b-null mice also weigh more than their WT counterparts after a HFD (Figure 1). WAT weight is usually associated with the increased body mass as determined by WSI. Old WT mice are the only group whose body mass rises at a greater rate than the measured WAT or WSI. Cyp2b-null mice fed a HFD or old Cyp2b-null mice showed further increases in WAT/WSI and liver weight/HSI compared to their WT counterparts (Figure 1). Interestingly, HSI went down in the HFD mice because of an increase in weight that was not concomitant with an increase in liver size. Only age caused increased liver weight in the B6 mice, which was also exacerbated by the lack of Cyp2b. In summary, age is associated with increased weight and WAT with similar trends following a HFD. A lack of Cyp2b exacerbated this observation.

**Figure 2:**
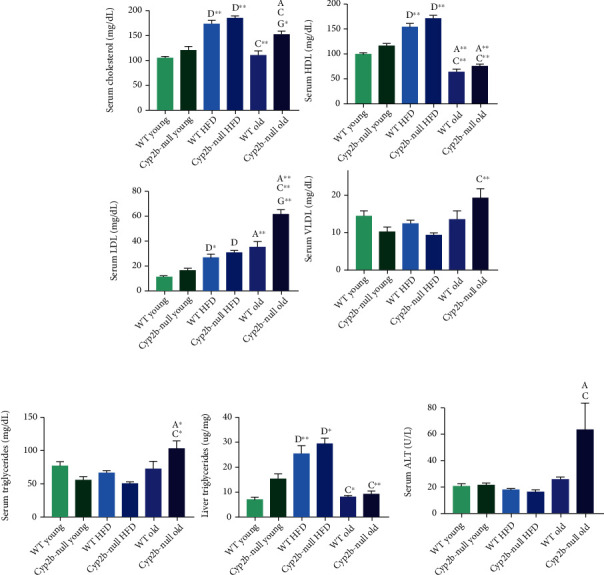
Perturbations in serum lipids, liver triglycerides, and alanine aminotransferase (ALT) with age, diet, and genotype. Serum cholesterol, HDL, LDL, and VLDL (a), serum and liver triglycerides (b), and serum ALT (c) were measured in all treatment groups using standard methods. Data are presented as mean ± SEM. Statistical significance was determined by one-way ANOVA followed by Tukey's multiple comparison test as the post hoc test (*n* = 5 − 6). “a” indicates age difference comparing young (4.5 mo) and old (9 mo) mice within the same genotype and diet group, “c” indicates difference between HFD-fed young (4.5 mo) and ND-fed old (9 mo) mice within same genotype, “d” indicates diet difference between ND-fed and HFD-fed mice within in same genotype and age, and “g” indicates genotype difference between WT and Cyp2b-null mice within same diet and age group. No asterisk indicates a *p* value < 0.05, ∗ indicates a *p* value < 0.01, and ∗∗ indicates a *p* values < 0.0001.

Serum cholesterol increased in old Cyp2b-null mice compared to old WT mice, but total cholesterol levels were highest in HFD-treated groups (Figure 2(a)). The relatively healthy HDL levels were highest following a HFD and exacerbated in Cyp2b-null mice. HDL significantly decreased as mice aged (Figure 2(a)). Diets high in *cis*-unsaturated fatty acids have also been found to increase HDL levels in humans in addition to serum cholesterol and LDL [42]. Conversely, LDL, VLDL (Figure 2(a)), and serum TAG (Figure 2(b)) levels were higher in old Cyp2b-null mice compared to all other groups. Age clearly had an adverse impact on these parameters. Liver TAG increased with diet but aging did not affect liver TAG compared to young mice (Figure 2(b)). Interestingly, alanine aminotransferase (ALT) (Figure 2(c)), a marker of liver damage, shows a spike with the combination of age and a Cyp2b-null genotype. Taken together, these results indicate a TAG-cholesterol profile, lower HDL, higher LDL, and VLDL that deteriorates with age and to a lesser degree, HFD. Often, this decline is greater in Cyp2b-null mice.

### 3.2. Hepatic Phospholipid Data Distribution and Perturbations by Age, Diet, and Loss of Cyp2b

**Figure 3:**
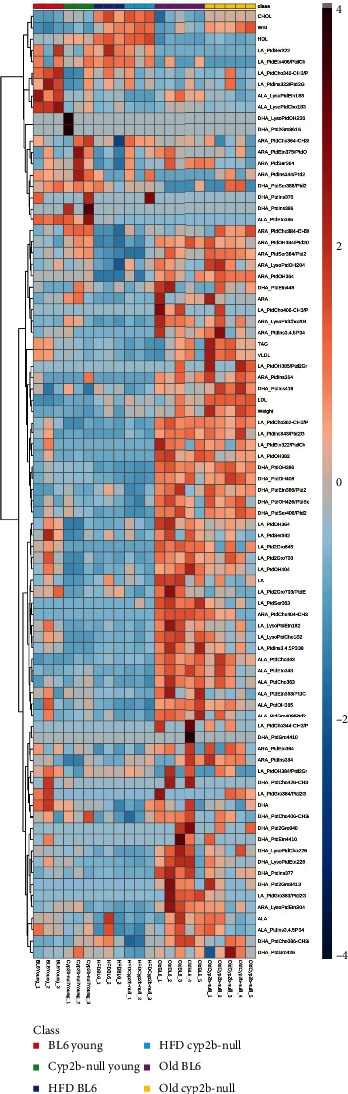
Heat map of phospholipid profiles of all treatment groups. Hierarchical cluster analysis heat map of all measured phospholipid species from ND-fed WT (BL6), ND-fed Cyp2b-null, HFD-fed WT, HFD-fed Cyp2b-null, old WT, and old Cyp2b-null mice. Seventy-seven lipid species were identified from liver samples of male mice by LC-MS/MS from arachidonic (ARA), linoleic (LA), *α*-linolenic (ALA), and docosahexaenoic acid (DHA).

Seventy-seven total hepatic phospholipid species were identified by LC-MS/MS from ARA, LA, ALA, and DHA. Hierarchical clustering was performed on all 77 lipid species identified to evaluate the effects of age, diet, and Cyp2b-null genotype on hepatic lipid species content (Figure 3 and data in Suppl File 1[43]). Age has a powerful effect on lipid profiles, more so than HFD or loss of Cyp2b based on the hierarchical cluster analyses (Figure 3).

### 3.3. Specific Lipid Species Are Associated with Adverse Physiological Events

**Figure 4:**
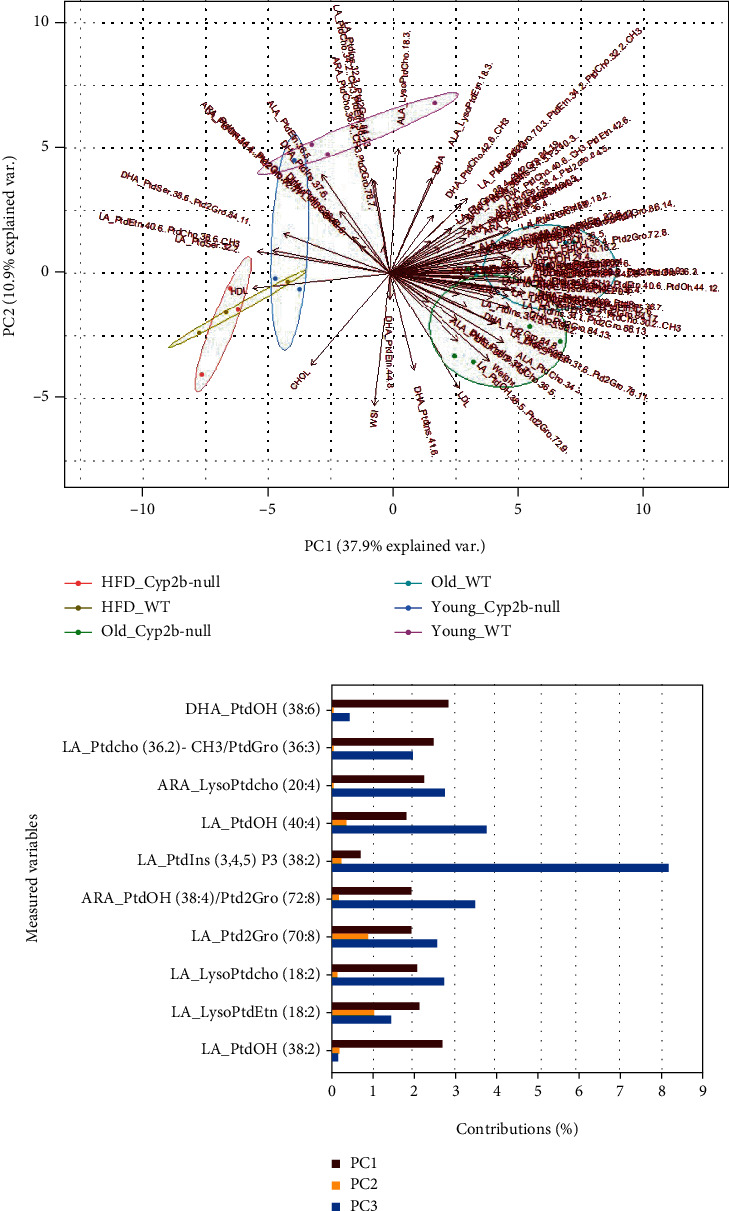
Relationship between treatment groups and measured variables. (a) Principal component analysis (PCA) biplot showing relationships between treatment groups and measured variables such as physiological parameters, serum lipids, and phospholipid species. Variables include serum lipids, WAT somatic index (WSI), body weight, and lipid species in order to associate specific biochemical parameters with different treatment groups (diet and age) and genotypes (WT and Cyp2b-null). (b) Top 10 contributing variables in the PCA plot based on loading strength expressed in percent contribution.

To determine associations between physiological parameters, serum lipids, and hepatic phospholipid profiles in the different treatment groups (genotype, age, and diet), PCA analysis was performed (Figure 4(a) and data in Suppl File 2 [43]). The 10 parameters that predominantly informed principle component differences are shown in Figure 4(b). These included predominantly ARA- and LA-based lipids with several being incorporated into PC, PE, or LPC. In general, treatment groups segregated primarily by age and diet; genotype caused lesser differences between groups as age in combination with genotype had a greater effect than a HFD in combination with genotype.

**Figure 5:**
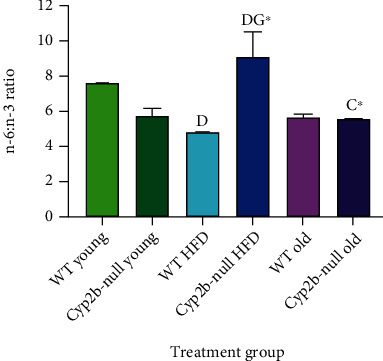
Changes in n-6 : n-3 ratios between treatment groups. Data are presented as mean (g) ± SEM. Statistical significance was determined by one-way ANOVA multiple comparison test with Tukey's as the post hoc test (*n* = 3 − 5). “a” indicates age difference between young (4.5 mo) and old (9 mo) mice within the same genotype and diet group, “c” indicates difference between HFD-fed young (4.5 mo) and ND-fed old (9 mo) mice within same genotype, “d” indicates diet difference between ND-fed and HFD-fed mice within in same genotype and age, and “g” indicates genotype difference between WT and Cyp2b-null mice within same diet and age group. No asterisk indicates a *p* value < 0.05, and ∗ indicates a *p* value <0.01.

ND-fed young Cyp2b-null mice were drawn towards the HFD-fed mice in the PCA plot, which is consistent with the increased triglycerides in this group (Figure 2). Similarly, significant changes in hepatic gene expression were observed previously in ND-fed Cyp2b-null compared to WT mice with these changes trending towards HFD-fed mice [29]. ND-fed young mice, especially WT, were associated with several specific n-3 (ALA/DHA) fatty acid species s in the PCA plots. More importantly, no adverse physiological or serum parameters were associated with young ND-fed mice (Figure 4), concurring with the previous data on the effects of ALA and DHA on healthy lipid homeostasis in humans [44, 45] and mice [46]. However, total n-6 : n-3 ratios were not lower in the ND-fed young mice. Surprisingly, they were lowest in the HFD-fed young WT mice, but highest in the HFD-fed Cyp2b-null mice, indicating the importance of diet and Cyp2b on fatty acid metabolism (Figure 5); HFD-fed young mice were associated with greater cholesterol, especially HDL and along with older mice showed an association with WSI (Figure 4). The lack of phospholipids in the HFD-fed mice quadrant is primarily caused by the dominance of higher phospholipid concentrations within the older mice (Suppl File 2, 3 [43]). Last, there was little separation between WT and Cyp2b-null mice following an HFD in contrast with the differences observed between WT and Cyp2b-null mice fed normal diets regardless of age.

Changes in distinct lipid species are associated with obesity or other adverse outcomes.

**Figure 6:**
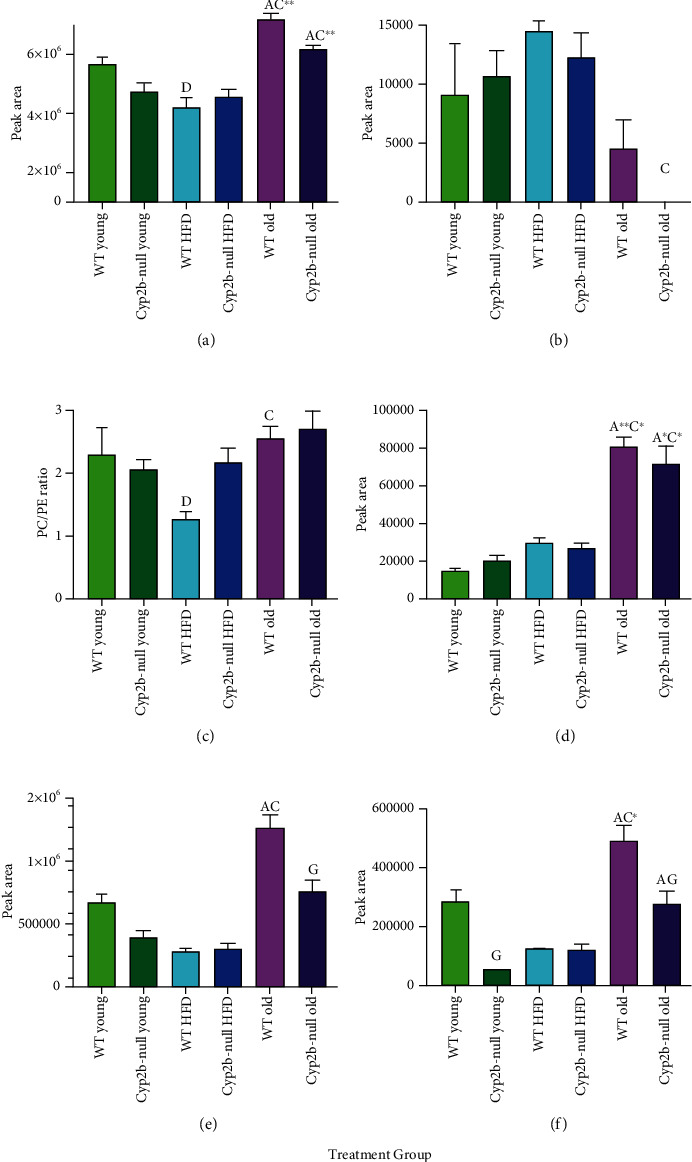
Comparison of distinct lipid groups or ratios between treatment groups. Total lipids, specific ratios, and lipid species were compared between all treatment groups: Total phospholipids (a), phosphatidylethanolamine 38 : 6 (b), total phosphatidylcholines/phosphatidylethanolamines ratio (c), phosphatidylcholine 34 : 3 (d), total lysophosphatidylcholines (e), and lysophosphatidylcholine 18 : 2 (f). Data are presented as mean (g) ± SEM. Statistical significance was determined by one-way ANOVA multiple comparison test with Tukey's as the post hoc test (*n* = 3 − 5). “a” indicates age difference between young (4.5 mo) and old (9 mo) mice within same genotype and diet group, “c” indicates difference between HFD-fed young (4.5 mo) and ND-fed old (9 mo) mice within same genotype, “d” indicates diet difference between ND-fed and HFD-fed mice within in same genotype and age, and “g” indicates genotype difference between WT and Cyp2b-null mice within same diet and age group. No asterisk indicates a *p* value < 0.05, ∗ indicates a *p*-value < 0.01, and ∗∗ indicates a *p* values < 0.0001.

Further evaluation of lipid groups, ratios, and species of interest associated with adverse outcomes showed some interesting patterns. Total phospholipids were significantly increased by age in both genotypes and decreased in WT-HFD relatively to WT-young (Figure 6(a)); in some cases, total phospholipids were inversely associated with liver triglycerides (Figure 2). PE 38 : 6, a plasma marker of aging [47], is clearly reduced in the old Cyp2b-null mice (Figure 6(b)). Liver PC/PE ratios are associated with liver disease [6]. WT-HFD mice demonstrated a clear drop in PC/PE ratio relative to WT-young mice and were also different than WT-old mice, indicating that the HFD had an effect on PC/PE ratio in the WT mice only (Figure 6(c)). Serum PC 34 : 3 is a putative biomarker of hypertension in patients with fatty liver diseases [48] and was increased with age (Figure 6(d)). Liver LPC levels are associated with liver damage [49]. Both LPC and LPC 18 : 2 were significantly increased by age and then decreased in older Cyp2b-null mice relative to WT mice (Figures 6(e) and 6(f)).

**Figure 7:**
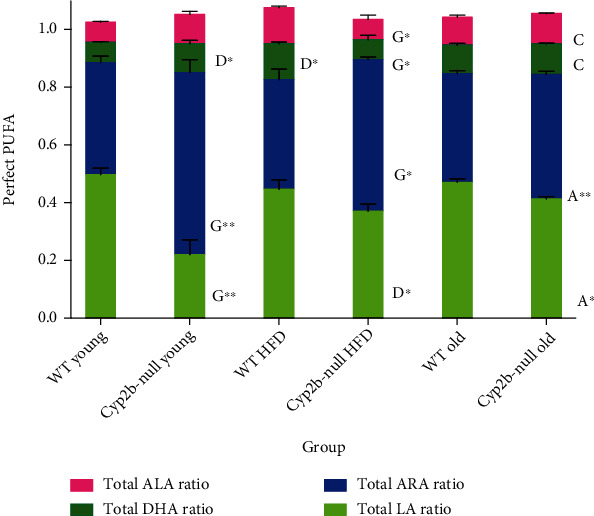
Ratio of each measured lipid (ALA, ARA, DHA, and LA) to total lipids. Data are presented as mean (g) ± SEM. Statistical significance was determined by one-way ANOVA multiple comparison test with Tukey's as the post hoc test (*n* = 3 − 5). “a” indicates age difference between young (4.5 mo) and old (9 mo) mice within same genotype and diet group, “c” indicates difference between HFD-fed young (4.5 mo) and ND-fed old (9 mo) mice within same genotype, “d” indicates diet difference between ND-fed and HFD-fed mice within in same genotype and age, and “g” indicates genotype difference between WT and Cyp2b-null mice within same diet and age group. No asterisk indicates a *p* value < 0.05, ∗ indicates a *p* value < 0.01, and ∗∗ indicates a *p* values < 0.0001. The ratio of each lipid is to the total (1.0) within a sample; however, average of each lipid type within a group varied, and therefore, the totals for each group do not necessarily equal 1.

Because total phospholipids varied by treatment group, we examined ratios between levels of each major PUFA measured: LA, ARA, ALA, and DHA. Many of the significant changes that occurred were genotype related, including a significant increase in ARA in young Cyp2b-null mice compared to young WT mice regardless of diet (Figure 7). Age reduced the ARA/LA ratio in old Cyp2b-null mice compared to young Cyp2b-null mice, which is considered a putative protective marker for liver disease, but positively associated with myocardial infarction [50]. Surprisingly, the relative amount of n-3 PUFA increased in HFD-fed WT mice relative to young WT mice and HFD-fed Cyp2b-null mice (*p* < 0.001). HFD-fed Cyp2b-null mice showed reduced n-3 PUFAs in comparison to old Cyp2b-null, young Cyp2b-null, and HFD-fed WT mice (*p* ≤ 0.01 for all three comparisons). In summary, changes in relative n-3 levels were not steady across diet and genotype. ARA concentrations were consistently increased relative to LA in young Cyp2b-null mice regardless of diet with a large decrease in relative LA levels in young Cyp2b-null mice.

### 3.4. Lipid Species and Outcomes Associated with Cyp2b-Null Mice

**Figure 8:**
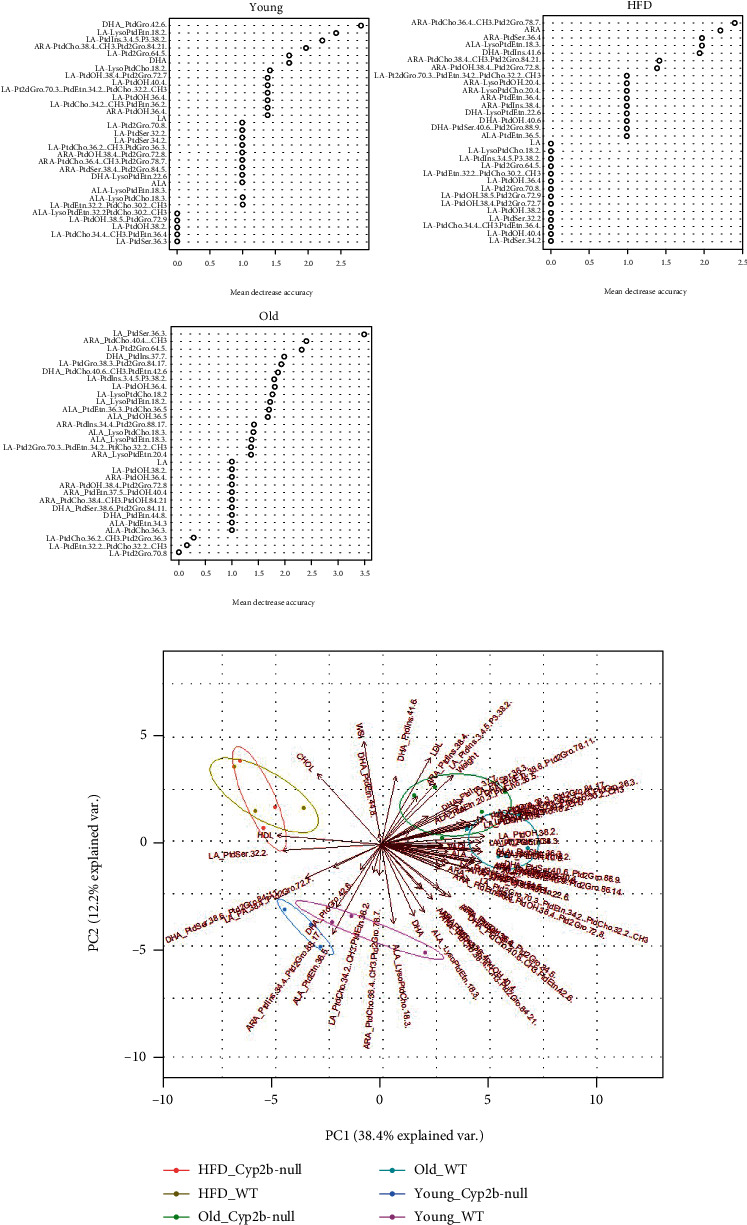
Lipid species and measured variables associated with differences between Cyp2b-null and WT mice. (a) Random forest analysis of key lipids predictive of differences between Cyp2b-null and WT mice. (b) Principal component analysis (PCA) biplot showing relationships between treatment groups and measured variables such as physiological parameters, serum lipids, and phospholipid species. Variables include serum lipids, WAT somatic index (WSI), body weight, and lipid species in order to associate specific biochemical parameters with different treatment groups (diet and age) and genotypes (WT and Cyp2b-null).

To better define the phospholipid species most predictive of differences between Cyp2b-null and WT mice, random forest was performed (Figure 8(a) and Suppl File 3-5 [43]). Very few lipid species were shared among the top 6 most predictive species between the different groups (young, old, and HFD) with the exception of ARA-PtdCho (38 : 4) (18 : 0-20 : 4 PtdCho (38 : 4)) found in ND- and HFD-fed young mice. LA species were common in the top 6 of ND-fed young and old mice; ARA species were much more common in the top 6 of HFD-fed mice when comparing WT and Cyp2b-null mice. This is not surprising considering the significant differences in ARA/LA ratios observed in the young Cyp2b-null mice (Figure 7). Most of the species that were different between WT and Cyp2b-null mice were decreased in the Cyp2b-null mice, potentially because of the absence of Cyp2b-mediated PUFA metabolism.

A PCA biplot was used to associate different hepatic phospholipid species, serum parameters, and physiological outcomes with age, diet, and Cyp2b status in the mice (Figure 8(b) and data in Suppl File 5a). This closer look at genotypic differences confirmed that despite few lipid species associated with young mice, some n-3 fatty acids along with a couple ARA and LA phospholipid species are associated with young ND-fed WT and Cyp2b-null mice, consistent with better health in these mice. n-6 : n-3 ratios were not as strong as predicted in the younger mice (Figures 5 and 7); however, individual n-3 species appear to make strong associations.

Fewer associated lipids and a relatively higher percent of them as n-3 may be a marker of health. ND-fed WT and Cyp2b-null mice show significantly different PCA profiles with no overlap potentially due to decreased metabolism; however, these ND-fed young groups are positioned next to each other within the plot. When considering the lipid species and variables that contributed the most to this Cyp2b status-based PCA plot, LA species made up 7 out of the 10 most dominant parameters, in addition to two ARA species and WSI (Suppl File 5 [43]).

## 4. Discussion

Old mice clearly had the unhealthiest serum profiles, strongest set of lipid species associations, and greatest difference between genotypes compared to ND-fed or HFD-fed young mice. The effect of age also superseded the effect of HFD on the blood lipidome of female mice [9]; therefore, our work further demonstrates the adverse effect of age on hepatic lipid profiles. Age was heavily associated with more fatty acids, larger fatty acids, n-6 fatty acids, and higher serum LDL, VLDL, and TAG concentrations. Age has also been found to increase total and LDL-cholesterol in both humans and rodents in the previous studies [51, 52]. In addition, increased hepatic phosphatidylcholine biosynthesis, as seen in the older mice, has been proposed to stimulate the production and secretion of VLDL and TAG [53]. Old Cyp2b-null mice were associated with weight and to a lesser extent WSI, while n-6 fatty acids showed a somewhat greater association with older WT mice. Greater dietary n-6 : n-3 ratios have been found to increase HDL levels without suppressing atherogenesis in mice [54]. Overall, old WT and Cyp2b-null mice are associated with unhealthy physiological, serum, and lipid profiles that contribute to an increased risk of metabolic disease and obesity [55, 56].

Several individual lipid biomarkers of metabolic disease were investigated from amongst the phospholipids measured. While there is significant information about disease associations with serum lipids, there have been few studies that associate specific liver phospholipids with metabolic disease, NAFLD, obesity, or aging. Serum PC/PE ratios are used as markers for potential liver disease with a lower ratio indicative of liver disease and a greater potential for NASH [36, 57]. However, recently murine liver studies indicate that higher ratios, primarily caused by higher hepatic PC concentrations cause increase VLDL, TAG, NAFLD, and NASH [6, 49, 53][6, 49, 53] Interestingly, liver and serum lipids are poorly correlated compared to liver and intestine, and in a few cases, liver and serum levels are inversely correlated [49]. PC/PE ratios trended higher with age, but did not provide a clear picture of disease state.

However, several other phospholipid biomarkers provided insight into the health of the mice. Serum LPC concentrations are typically inversely associated with obesity [58], and specifically, LPC 18 : 2 is inversely associated with childhood obesity, BMI, and metabolic risk factors in humans [48, 58, 59]. Conversely, recent research with murine livers indicates that greater liver LPC concentrations are associated with NAFLD [49], but inversely associated with NASH [6]. It is possible that reduction of serum LPC may be at the expense of liver LPC. Hepatic LPC and LPC 18 : 2 were significantly increased by age (Figure 6(e)). LPC 18 : 2 was also lower in Cyp2b-null young mice compared to WT young mice (Figure 6(f)); however, the interpretation of this data depends on future work and its associations with serum LPC 18 : 2 as to whether lower levels suggest protection. Overall, our LPC data suggests that aging is negatively affecting the liver and NAFLD may follow. Serum PE 38 : 6 and PE 34 : 3 are associated with longevity and hypertension, respectively [47, 48]. Both indicate the adverse effect of aging; however, it is possible that these liver markers are not correlated with the serum markers. Taken together, phospholipid biomarkers indicate significant liver damage in older mice with probable increases in hypertension, liver damage, and shorter lifespan. Cyp2b-null mice show markers consistent with lower liver damage but also reduced lifespan.

When examining parameters based strictly on differences in Cyp2b status by PCA, HFD-fed mice show greater separation from ND-fed mice in comparison to analyses with all parameters conducted together. This is primarily because the lipid profile of ND-fed Cyp2b-null mice clustered between ND-fed WT mice and HFD-fed groups, consistent with their liver triglyceride concentrations. HDL levels were also significantly higher in HFD mice, whose diet provided nearly ten times more n-6 than n-3 PUFAs. Greater HDL was also observed in Cyp2b-null mice than WT mice regardless of diet; no differences in HDL were observed between genotypes in older mice. In addition, changes in fatty acid chain length were not observed in Cyp2b-null mice, as observed in HFD-fed Cyp3a-null male mice [60]. Older mice demonstrated more differences between genotypes with a higher association with weight, WSI, and LDL in Cyp2b-null mice and more phospholipids clustering with old WT mice. In general, findings are consistent with lower metabolism of some PUFA species in Cyp2b-null mice, which was also observed in hepatic P450 reductase- and Cyp3a-nullizygous mice [60, 61].

Low expression of human CYP2B6 is associated with obesity. It is the only detoxification CYP to be associated with obesity in humans [30]. Furthermore, CYP2B enzymes show lower expression in rats as they age [62], and CYP2B6 shows lower expression in humans as they age [63]. Higher expression of Cyp2b enzymes is also associated with longevity in dwarf mice [64]. Therefore, age-dependent loss of CYP2B enzymes could affect lipid metabolism, perturb lipid depuration, and increase aging. There are also some key CYP2B6 polymorphisms that effect drug metabolism with CYP2B6∗4, CYP2B6∗5, and CYP2B6∗6 the most common [65, 66]. In turn, these variants show clinically significant adverse outcomes because of slow or ineffective metabolism, including metabolism of propofol, efavirenz, BDE-47, ketamine, and bupropion [67–72]. Ketamine also shows reduced metabolism and clearance in older humans [63]. The effect of age or CYP2B6 polymorphisms on lipid metabolism has not been studied.

In summary, the data indicates that age > HFD > Cyp2b-null genotype compromises the hepatic phospholipid profile the most with lipid profiles and other parameters providing biomarkers of health status. ND-fed old Cyp2b-null and HFD-fed young mice show significant changes in several phospholipids and physiological parameters such as serum cholesterol and WAT compared to ND-fed young mice, which have significantly lower lipids. Interestingly, the lipid profile of ND-fed Cyp2b-null mice clustered between ND-fed WT mice and HFD-fed groups, indicative of increasing lipid concentrations and reduced health in ND-fed Cyp2b-null mice even without additional fats in their diet. Total body and liver weight, serum LDL, VLDL, TAG, and ALT levels are all significantly higher in old Cyp2b-null mice compared to their WT counterparts as well as several featured groups. The combination of age and lack of Cyp2b is more harmful than age alone, as it resulted in dyslipidemia and liver injury. Overall, aging and a HFD are associated with weight gain, WAT, LDL, and VLDL; however, global n-6 : n-3 ratios are only affected in the HFD group, and DHA/LA ratios were perturbed by the loss of Cyp2b. Overall, phospholipidomic profiles are age-dependent > diet dependence and further exacerbated in Cyp2b-null mice, suggesting accelerated aging or metabolic disease symptoms with the lack of Cyp2b in male mice.

## Figures and Tables

**Table 1:** Solvent gradient for resolution of polar lipids.

Gradient		
Time	%A	%B
Initial	80	20
1.00	80	20
2.10	60	40
8.00	30	70
10.00	30	70
12.00	0	100
14.00	0	100
14.10	80	20
15.00	80	20

Solvent A: 40 : 60 water/acetonitrile. Solvent B: 90 : 10 isopropanol/acetonitrile.

**Table 2:** Mass spectrometry instrumental parameters during resolution of polar lipids.

Curtain gas (CUR)	25.00		Collision gas (CAD)	Low
ESI voltage (IS)	-3500		Gas source 1 (GS1)	55.00
ESI temp (TEM)	650°C		Gas source 2 (GS2)	50.00
Declustering potential	-90.00		Collision energy (CE)	-40.00
Entrance potential (EP)	-10.00		Collision energy spread	0.00
Q1 and Q3 resolution	Unit		Step size	0.2 Da

## Data Availability

All data is within the manuscript or provided in the supplementary files [44].

## Abbreviations

ALT: Alanine aminotransferase
ALA: *α*-Linolenic acid
ARA: Arachidonic acid
CYP: Cytochrome P450
DIO: Diet-induced obesity
DHA: Docosahexaenoic acid
HNF4*α*: Hepatic nuclear factor 4*α*
HIS: Hepatosomatic index
HDL: High density lipoprotein
HFD: High-fat diet
LA: Linoleic acid
ALA: *α*-Linolenic acid L
LXR: Liver X receptor
LDL: Low density lipoprotein
LPC: Lysophosphatidylcholine
MDA: Mean decreased accuracy
NAFLD: Nonalcoholic fatty liver disease
NASH: Nonalcoholic steatohepatitis
ND: Normal diet
OOB: Out of bag
PUFA: Polyunsaturated fatty acid
PPAR: Peroxisome proliferator-activated receptor
PC: Phosphatidylcholine
PCA: Principal component analysis
PE: Phosphatidylethanolamine
VLDL: Very low density lipoprotein
WAT: White adipose tissue
WSI: White adipose somatic index
WT: Wild-type.

## Supplementary Materials

Supplementary Materials Supplementary File 1 Lipidomic data from all treatment groups. Seventy-seven lipid species were identified from liver samples of male mice by LC-MS/MS from arachidonic (ARA), linoleic (LA), *α*-linolenic (ALA), and docosahexaenoic acid (DHA). Treatment groups are ND-fed young (4.5 mo), ND-fed old (9 mo), and young mice fed a HFD for 10 weeks. All data is in peak area. Supplementary File 2 Data used to make Figure 4 principal component analysis (PCA) biplot. Supplementary File 3 (a) Lipids that are statistically different by Student's *t*-tests between Cyp2b-null and WT mice in the different age and diet groups. (b) Important or significantly perturbed phospholipid species in Cyp2b-null mice compared to WT mice identified by random forest analysis and Student's *t*-tests in young (4.5 mo) ND-fed, young (4.5 mo) HFD-fed, and old (9 mo) mice. Supplementary File 4 Important perturbed hepatic phospholipid species in Cyp2b-null mice within each group. Venn diagram of important lipid species between Cyp2b-null and WT mice of each featured group based on random forest analysis (MDA>0). Supplementary File 5 (a) Data used to make principal component analysis (PCA) biplot for Figure 5(b). (b) PCA contribution graph below containing the top 10 contributing variables of the PCA plot based on loading strength expressed in percent contribution. The factoextra R package (https://cran.r-project.org) was used to obtain the percent contributions of each measured variable in principle components 1-3.
